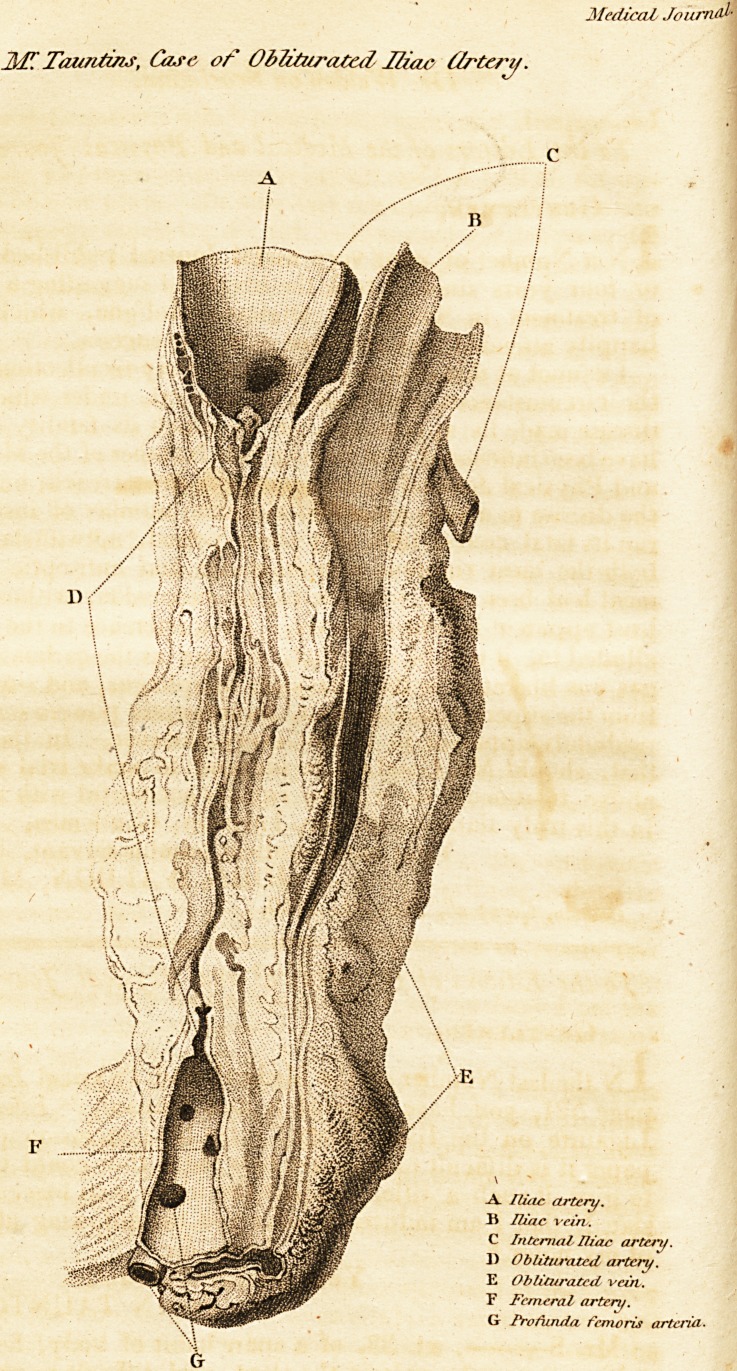# Case of Obliterated Iliac Artery

**Published:** 1811-01

**Authors:** John Taunton


*To the Editors of the Medical and Physical Journal.*

GENTLEMEN,

*To the Editors of the Medical and Physical Journal.*

GENTLEMEN,

IN the last Number of the Medical and Physical Journal, page 524, you have inserted a case entitled, "Effect of a Ligature on the Iliac Artery." By the conclusion of this paper it is difficult to conceive how the author could venture to give it such a title. Indeed the whole is so inaccurately stated, that I am induced to offer you the following abstract of my notes.* I am, &c.

Your obedient Servant,

JOHN TAUNTON.

Mr. S———, æt. 33, of a spare habit of body, has been subject to cough, pain in the chest, and difficult respiration,

* The case alluded to by Mr. Taunton, was copied *verbatim* from the last Number of the London Medical Review.

for several winters, which symptoms generally disappeared during the summer months.

There had been an enlargement of the left testicle for upwards of four years. In May last the operation of castration was thought necessary by the professional gentlemen under whose care he had been. I was permitted to be present at the operation.

In passing the knife through the integuments from the abdominal ring towards the depending part of the tumor, some cists were cut into at the upper part of the testicle. From these a limpid fluid escaped, (probably about two or three ounces.) The integuments being separated from the sides of the tumor, and spermatic process, the latter was then divided. Several attempts were made to take up the spermatic arteries with the tenaculum, but the ligatures had no effect in suppressing the hæmorrhage, probably on account of the retraction of the spermatic arteries within the cellular substance of the cord. The spermatic process (which was very short from the great enlargement of the testicle) receded within the abdominal ring, the incision was then enlarged upwards with a view of exposing the arteries above the external ring. These attempts failing, the bleeding, which was very profuse, could only be restrained by passing a large needle, armed with a very strong ligature, high up under the spermatic process, and enclosing within the ligature the whole surface from whence the hæmorrhage issued.

This succeeded completely with regard to the spermatic arteries; some smaller vessels from the cellular substance were secured, and the patient put to bed in a very weak state from great loss of blood during an operation of nearly two hours.

In the night there was a considerable hæmorrhage from the vessels of the cellular substance; this was stopped by powdered ice laid on the part with tow.

From the 25th of May to the 14th of June, I did not visit the patient, but was informed by the gentleman who attended him, that febrifuge medicines were given for some days after the operation. Afterwards a more nutritious diet was allowed and bark given; that the ligatures had all come away, that the wound looked healthy, and was healing fast on the scrotum.

On the 14th of June a small swelling appeared at the upper part of the wound, which was painful; a poultice was applied. About eleven P. M. the swelling burst, from the opening of which a large stream of arterial blood issued.—Lint and cloths moistened with cold water were constantly applied.

applied to the wound with pressure, and succeeded partially in stopping the hæmorrhage.

Between eleven and twelve P.M. I saw him for the first time after the operation, and found him very low from the loss of arterial blood, which had been considerable; but at this time there remained only an oozing from the coagula, lying at the upper part of the wound.—R. liq. plumbi acetat. ℥ij. aquæ ℥vj. M. Lint and cloths moistened in the above lotion were applied to the wound, with directions to make pressure with the hand if the bleeding should return; the room was kept cool, and the patient ordered to be perfectly quiet.

June 15, 16, 17, and 18. Bandages were applied round the body to produce different degrees of pressure on the part; but without any apparent advantage, as the bleeding returned at short intervals.

19th. A steel truss was applied with a screw to the pad, by which the pressure could be greatly increased; the pad of the truss was large, and applied immediately over the course of the external iliac artery, the lower part reaching to the edge of Poupart's ligament.—This produced great pain, from the pad imbedding itself in the wound, without completely restraining the hæmorrhage, which returned several times during the 20th, 21st, and 22d; rather profusely on the latter day.

The pad of the truss had, by the 22d, completely embedded itself in the soft parts, producing great constitutional irritation.

23d. At eight A.M. the hæmorrhage had been profuse; the stream of blood very large, and evidently arterial: it could not be stopped by pressure.

Five A.M. It was now agreed in consultation to remove the truss, and if possible to expose and secure the bleeding artery.

Pressure was made upon the primitive iliac artery, and the truss removed. The pad had destroyed the integuments, muscles, and tendons, and completely embedded itself in a large cavity extending nearly to the iliacus internus muscle. The surface of the cavity was a confused mass in a sloughing state.*

The epigastric artery appeared ulcerated near its origin, having been destroyed by the pressure of the truss.—An in-

* The author of the paper in your last Journal is so little acquainted with the real history of the case, that he speaks of the hæmorrhage as occurring "at the expiration of some weeks from the application of the truss." He ought to have informed himself that the truss only remained on between *four* and *five days*.

cision cision of about two inches in length was made through the integuments and muscles, from the upper surface of the wound towards the superior margin of the ilium in the direction of the fibres of the external oblique muscle.—Little hæmorrhage followed the complete exposure of the wound. A ligature was now made on the iliac artery, but still some bleeding was observed to come from a small vessel in the direction of the spermatic artery. This was taken up with the tenaculum. The ligature which had been passed on the iliac artery was removed, at which time a tingling sensation was experienced down the thigh in the course of the crural nerve: one suture was passed in that part of the wound where the incision had been made. The wound was dressed with lint moistened in a lotion of some water, acid, and camphor.

The wound suppurated kindly; the discharge was very considerable; the contused parts separated; the ligature came away in a few days; the granulations appeared healthy, excepting in one part immediately over the iliac artery, where a kind of downy appearance was seen on the granulations, which were of a pale colour. This disappeared gradually; the wound filled up with healthy granulations, and at the end of three weeks required to be dressed only every second day, the lower part being now completely healed.

During this period a nourishing diet was allowed, some bark had been taken in Port wine, and occasionally an aperient medicine, the general health and strength were much improved, so that on the 15th of July, the patient could be raised in the bed, and held up in nearly a sitting posture for some time. This change of position was repeated several times on the 16th, and seemed to give additional strength, the wounds on the back and lips had become very painful, from lying so long in one position. On the 17th he was lifted out of bed and placed in a chair, the body inclined forwards on a pillow laid on a table.

This change of position agreed well, and was repeated two or three times each day; the general health was much improved, but some swelling of the legs took place, particularly about the ancles; pain was felt down the inside of the thigh in the course of the Sartorius muscle; there was also an irregular, hard, knotty line, extending in the same direction, very painful to the touch; this line was longest and most painful near the groin.

The wound continued to heal and look healthy, the hardness and pain on the inside of the thigh gradually subsided. A nutritious regimen, bark infused in wine, and aperients were continued; when his cough (to which he was very liable)

ble) was troublesome, he took misturæ ammoniaci cum tinct. digitalis.

On the 5th of August he appeared as well or rather better than usual, but I observed that the granulations looked pale, and that the edges of the wound had an irregular appearance, which had not been seen before; he sat up near ten hours during this day, went to bed as well as usual, and had a tolerable night.

On the 6th, soon after he had been lifted out of bed, the wound began to bleed, but not profusely; he was immediately put to bed again, lint and cloths moistened in cold water were applied to the wound; this succeeded in stopping the hæmorrhage, and the qnantity of blood lost (which was arterial) was not more than from 4 to 8 ounces. On removing the cloths and lint there was a small coagulum at the bottom of the wound immediately over the iliac artery, at least two inches from the part where the ligature had been applied on the small artery on the 23d June. Pledgets of lint moistened in a similar lotion to that ordered on the 14th of June were applied to the wound, he was laid on his back, the knees raised. No bleeding during the remainder of this day. In the evening, on removing the lint the coagulum was separated and a small sinus observed at the bottom of the wound over the iliac artery. A probe introduced about an inch deep in a direct line to the artery had a distinct pulsating motion communicated to it.

The pulsation in the femoral artery through its *whole course* had continued the same as in the other thigh during the *whole* confinement.

August 7. He had slept a little during the night, but suffered much this morning from the position of the body, which had not been changed, he had also great pain in his left side with difficult respiration.

Bottles of warm water were ordered to be applied to his side, and a mixture of the liquor ammoniæ acetat. cum syrup. papav. alb. to be taken frequently.

August 8. From this time to the 26th September the original wound often granulated in a healthy manner, and even seemed disposed to cicatrize, yet his general health gradually declined, he became hectic and even anasarcous, with those deceitful intermissions which so often occur in these cases. The wounds in his back assumed an unfavourable aspect, and his cough and expectoration encreased. From the 26th to the 30th he became much worse, respiration was short and painful, the granulations looked flabby and pale, and the edges a little irregular, the hands were somewhat swelled.

October 1st. Much worse, respiration very short and painful, the countenance indicated a speedy dissolution.

October

October 2d.—2 A. M. After a restless night he is a little revived, but in the whole much worse than he was yesterday. —Died at 1 P. M.

*Examination after Death.*

October 3d. The abdominal viscera were healthy, excepting some adhesions between the intestines and the peritoneum: in the left inguinal and iliac regions, the peritoneum, iliac artery and vein were united by a dense substance, evidently the effects of extensive and active inflammation.—The iliac artery was obliterated from within half an inch of the internal iliac to the under part of Poupart's ligament about $3\frac{1}{4}$ inches in extent.—The arteria profunda was given off, high up, near Poupart's ligament, and neither that nor the femoral artery appeared to have undergone any change at that part.

What appears to me to deserve particular attention is, that the pulsation of the femoral artery at the under edge of Poupart's ligament, and below, never suffered any change in strength or frequency from that which was observed in the artery of the right thigh.

The iliac vein was obliterated from a little above Poupart's ligament for one inch and half downwards, which was the part where the vessel was divided, hence it could not be ascertained how low the obliteration was continued.

Is it not probable that the obliteration extended along the femoral vein, and that the pain, and the irregular knotty line, which was observed on the 17th of July, and following days, in the direction of the Sartorius muscle, were occasioned by the obliteration of this, and the Saphenæ major veins?

The experiments of Dr. Jones tend to prove, that the obliteration of an artery must be preceded by inflammation. Whether this inflammation is produced by injury done immediately to the artery itself, or to the surrounding parts, and from them communicated to the artery, cannot be of importance, if, as Dr. Jones supposes, the whole effect is produced by inflammation. In this instance there cannot be any reasonable grounds for attributing the obliteration to the ligature, especially as the iliac and femoral veins were obliterated without any ligature, and also as the inflammation, ulceration, and destruction of parts produced by the pressure of the truss in the neighbourhood, and upon the obliterated vessels, were much more than sufficient to explain the event. It is very far from my wish to undervalue Dr. Jones's labours, or the conclusions he draws from them, they are supported by experiments, and, in my opinion, would not receive aid from any inference drawn from the preceding case.

*Memoir*

## Figures and Tables

**Figure f1:**